# Role of tight junction proteins in gastroesophageal reflux disease

**DOI:** 10.1186/1471-230X-12-128

**Published:** 2012-09-20

**Authors:** Klaus Mönkemüller, Thomas Wex, Doerthe Kuester, Lucia C Fry, Arne Kandulski, Siegfried Kropf, Albert Roessner, Peter Malfertheiner

**Affiliations:** 1 Department of Gastroenterology, Hepatology and Infectious Diseases, Otto-von-Guericke University, Magdeburg, Leipziger Str. 44, Magdeburg, 39120, Germany; 2 current address: Department of Gastroenterology, Hepatology, and Infectious Diseases Marienhospital, Bottrop, 46236, Germany; 3 Institute of Pathology, Otto-von-Guericke University, Leipziger Str. 44, Magdeburg, D-39120, Germany; 4 Institute of Biometrics and Medical Informatics, Otto-von-Guericke University, Leipziger Str. 44, Magdeburg, D-39120, Germany

**Keywords:** Gastroesophageal reflux disease, Tight junction, Claudins, Esophagitis, Inflammation

## Abstract

**Background:**

Gastroesophageal reflux disease (GERD) is associated with impaired epithelial barrier function that is regulated by cell-cell contacts. The aim of the study was to investigate the expression pattern of selected components involved in the formation of tight junctions in relation to GERD.

**Methods:**

Eighty-four patients with GERD-related symptoms with endoscopic signs (erosive: n = 47) or without them (non-erosive: n = 37) as well as 26 patients lacking GERD-specific symptoms as controls were included. Endoscopic and histological characterization of esophagitis was performed according to the Los Angeles and adapted Ismeil-Beigi criteria, respectively. Mucosal biopsies from distal esophagus were taken for analysis by histopathology, immunohistochemistry and quantitative reverse-transcription polymerase chain reaction (RT-PCR) of five genes encoding tight junction components [Occludin, Claudin-1, -2, Zona occludens (ZO-1, -2)].

**Results:**

Histopathology confirmed GERD-specific alterations as dilated intercellular spaces in the esophageal mucosa of patients with GERD compared to controls (*P* < 0.05). Claudin-1 and −2 were 2- to 6-fold upregulation on transcript (P < 0.01) and in part on protein level (P < 0.015) in GERD, while subgroup analysis of revealed this upregulation for ERD only. In both erosive and non-erosive reflux disease, expression levels of Occludin and ZO-1,-2 were not significantly affected. Notably, the induced expression of both claudins did not correlate with histopathological parameters (basal cell hyperplasia, dilated intercellular spaces) in patients with GERD.

**Conclusions:**

Taken together, the missing correlation between the expression of tight junction-related components and histomorphological GERD-specific alterations does not support a major role of the five proteins studied in the pathogenesis of GERD.

## Background

Gastroesophageal reflux disease (GERD) is one of the most prevalent gastrointestinal disorders in the world [1,2]. Based on endoscopic findings GERD is differentiated in erosive (erosive reflux disease or ERD), non-erosive reflux disease (NERD) and Barrett’s esophagus (BE) [3,4]. ERD is characterized by endoscopic visible breaks of esophageal mucosa integrity and classified according to various endoscopic classifications, most recently the Los Angeles classification [5,6]. However, two thirds of patients with typical GERD symptoms do not exhibit visible mucosal changes in conventional esophagogastroduodenoscopy (EGD) and are thus diagnosed as having NERD [6,7]. Although histology is not used in clinical practice for GERD diagnosis, frequent histological changes as basal cell hyperplasia, elongation of the papilla, inflammatory infiltrates and dilatation of the intercellular spaces are observed in the distal esophagus of patients with both ERD and NERD [8-11]. Dilations of the intercellular spaces (ICS) are characteristic changes of the esophageal mucosa of patients with ERD and NERD. ICS were described by various others using electron microscopy and are even characterized by light microscopy. This feature is being more widely proposed as an additional morphological feature of acid-induced damage to the squamous epithelium [10,12-14]. The widened ICS are supposed to permit the diffusion of molecules to the lamina propria where sensory nerve endings are located [15]. Therefore, ICS dilation even in the absence of endoscopically visible mucosal damage may explain the occurrence of symptoms in patients with NERD [16,17]. Furthermore, recent studies have provided evidence that the impaired barrier function of esophageal mucosa is a “hallmark” of GERD [18-20]. The integrity of epithelial surfaces is based on various cell-cell contacts that provide the structural basis for barrier function by regulating the diffusion of molecules and sorting of transmembrane proteins to apical and basolateral surfaces. Tight junctions, adherens junction and desmosomes are the three major structural units mediating barrier and sorting function [21,22]. Their structural composition, general functions, and pathophysiological relevance have been reviewed extensively by others [21,23,24]. In line with the current concept in GERD, the role of molecules contributing to cell-cell contacts in esophageal mucosa in relation to GERD has been investigated in animal and human studies recently. Notably, the majority of studies were focused on the role of tight junction molecules (e.g. Claudin-2, -3, -4, -7 and −18) in Barrett’s metaplasia and carcinogenesis towards esophageal adenocarcinoma [25-30]. In regard to the other 2 endoscopic entities (ERD, NERD), distinct alterations in the expression and or localization were described for Claudins 3 and 4 in GERD-related animal and *in vitro* models [31-33]. Rat model revealed decreased expression of Claudin-3 and no change of Claudin-1 and 4 [31,32], while an *in vitro* model of esophageal-like squamous cells demonstrated a prominent role of Claudin-4 [33].

Here, we studied the expression patterns of five tight-junction related molecules (Occludin, Claudin-1, -2 and Zonula occludens-1-, 2) in the esophageal mucosa of a prospective cohort of patients with GERD as well as reflux-negative individuals. Gene expression was assessed both on transcriptional and protein level, and changes were studied in context to histopathological alterations associated with GERD.

## Methods

### Study design and patients’ characteristics

Between 2005 and 2007, a cohort of patients with GERD and individuals lacking any symptom or endoscopic sign of GERD as GERD-negative controls were enrolled [34]. Patients with typical GERD-related symptoms based on Montreal classification [4] and patients without any reflux-related clinical symptoms undergoing EGD for screening or non-reflux dyspepsia (GERD-negative controls with a reflux disease questionnaire, RDQ score of 0) were invited to participate. All the patients underwent a detailed history and physical examination. The demographic data and endoscopic findings of the study population are presented in Table 1A and Table 1B. Written informed consent was obtained from all patients before endoscopy, after the endoscopist had explained the procedure to the patient in detail and answered all questions. The study was approved by the ethical committee of our institution and conducted according to the ethical guidelines of the declaration of Helsinki as revised in 1989.

**Table 1:** Patient groups analyzed by quantitative RT-PCR and immunohistochemistry

**Quantitative RT-PCR**	**Controls ** **(n = 26)**	**NERD ** **(n = 37)**	**ERD ** **(n = 47)**
*Sex (male/female)*	6/20	6/31	31/16^#^
*Age (mean, sd, range)*	52.3 ± 17.6	47.0 ± 14.1	47.5 ± 15.4
	(20–79)	(18–72)	(20–79)
*H. pylori-status (positive)*	5/21	7/30	12/35
	(23.1 %)	(22 %)	(29.2 %)
**Immunohistochemistry**	**Controls (n = 12)**	**NERD (n = 13)**	**ERD (n = 16)**
*Sex (male/female)*	2/10	4/9	10/6^#^
*Age (mean, sd, range)*	46.2 ± 19.1	48.9 ± 9.5	48.6 ± 14.1
	(20–75)	(35–64)	(29–72)
*H. pylori-status (positive)*	4/8	2/11	7/9
	(33.3 %)	(15.4 %)	(43.8 %)

Functional investigations such as 24 hour-pH-metry or MII-pH analysis were performed in individual cases only, and could not be included as separate parameter. The assignment of NERD was additionally based on the responsiveness to PPI therapy that was subsequently assessed.

#### Inclusion criteria

Female or male, age 18 to 80, able to provide written informed consent. Patients with typical reflux symptoms had to present symptoms at least three times a week. Typical reflux symptoms were defined as heartburn and regurgitation, as evaluated by the RDQ score. Patients with other types of reflux symptoms were not included in this study.

#### Exclusion criteria

Upper gastrointestinal pathology (e.g. peptic ulcers, cancers, polyps, and Barrett’s mucosa), systemic inflammatory, neoplastic or malabsorptive diseases (e.g. Crohn’s disease, ulcerative colitis, vasculitis, celiac disease), and acute medical conditions such as pneumonia, stroke, coronary ischemia and acute renal failure. Patients with known abnormal coagulation parameters and thrombocytopenia at the time of the procedure (i.e. INR > 1.2, platelet count < 80,000) were also excluded. None of the patients had taken antibiotics, or bismuth compounds or any H2-blockers or proton-pump inhibitors (PPI) in the last 2 weeks before entering the study. It is notable that the majority of patients enrolled had various anti-secretory medications in their past, and does not present GERD-naïve patients. Each patient was assigned a coded number. Histopathological assessment was done by pathologist (DK) blinded to clinical data.

### Endoscopy and histopathology

The patients underwent the procedure after an overnight fast. The endoscopy was performed under conscious sedation with intravenous midazolam using a videogastroscope (Q160, Olympus, Hamburg). Endoscopic characterization of esophagitis was performed according to the “Los Angeles classification” [35] describing the following endoscopic landmarks: gastroesophageal junction (GEJ), Z-line, beginning of the gastric folds and diaphragmatic pinch. The GEJ was defined as the beginning of the gastric folds, whereas the Z-line was defined as the squamocolumnar junction. The cardia was defined as the mucosa lying immediately below the GEJ.

In the distal esophagus, 3 biopsies were taken 2 cm above the squamous-columnar junction at the 3 o'clock position. In case of erosions, specimens were taken 2 cm above the tip of the erosion. One biopsy was snap-frozen in liquid nitrogen for molecular analysis. The two other biopsies were immediately fixed in 4 % neutral-buffered formalin and submitted for histopathological examinations using hematoxilin and eosin, modified Giemsa and PAS stain. In analogy to the Sydney classification for gastritis, the density of intraepithelial neutrophils/eosinophils and lymphocytes were scored to evaluation active and chronic inflammation. Furthermore, degree of basal cell hyperplasia, presence of papillary elongation and dilated intercellular spaces were semiquantitatively scored as either 0 (absent), 1 (mild), 2 (moderate), or 3 (severe) as described previously [34]. Notably, several subgroups of the study cohort were published in regard to inflammatory mediators (e.g. cytokines, Protease-activated receptor 2) [34,36], molecules related to barrier functions [37,38], desmosomal proteins [39] and histopathological alterations [34].

### Extraction of RNA and quantitative reverse transcription - polymerase chain reaction (RT-PCR) analysis of tight junction-related genes

Extraction of total RNA and cDNA synthesis were performed by the “two-step” protocol as described previously [40]. Transcript levels of Occludin, Claudin-1, -2, Zonula occludens-1, -2, and β-Actin were determined by quantitative real-time RT-PCR using an iCycler (BioRad, Munich, Germany) and the QuantiTect™ SYBR Green kit (Qiagen) using primers and standard conditions described in Table 2. Initial template mRNA amounts for all genes were calculated using iCycler software (Ct-values) and serial dilutions of plasmid DNA standard containing the corresponding PCR-fragments. Calculating template concentrations based on the Ct method and standard dilutions allowed an individual assessment of different efficiency for each PCR assay that were between 0.95 and 0.99. Gene-specific levels were normalized to the corresponding ß-actin level of the sample. Final results are expressed as arbitrary units (a.u.) and represent ratios between investigated gene and ß-Actin transcript amounts. All together, gene expression levels are identical to those calculated by the 2-∆∆ Ct-method [41], but they are additionally adjusted to the assay-specific efficiency. Due to the primer design (usage of intron-spanning regions), amplification of genomic DNA was excluded. All amplification products were checked for their correct size by agarose gel electrophoresis. Therefore, gene expression levels (a.u.) illustrate the mRNA pool of the individual gene studied.

**Table 2:** Characteristics of primers, RT-PCR protocol and antibodies

	**Primer sequence, length of fragment, annealing temperature**	**Antibody, Company, Antigen retrieval, final dilution**
***Occludin***	fw: GGCCATTGCCATTGTACTGGG rv: GGAACCGGCGTGGATTTATAGG 315 bp; 58°C	polyclonal rabbit anti-occludin antibody No. 71–1500 (Invitrogen, Carlsbad, CA, USA), Protease-retrieval, Final dilution: 1:50
***Claudin-1***	fw: ATGGTGGTTGGCATCCTCCTG rv: GGCCTTGGTGTTGGGTAAGAGG 344 bp, 58°C	polyclonal rabbit anti-Claudin-1 antibody No. 51–9000, clone JAY.8 (Invitrogen, Carlsbad, CA, USA), EDTA-retrieval, Final dilution: 1:50
***Claudin-2***	fw: TCTCTTGGCCTCCAACTTGTGGG rv: GCACTGGATGTCACCATCATGGC 259 bp, 60°C	polyclonal rabbit anti-Claudin-2 antibody No. 51–6100 (Invitrogen, Carlsbad, CA, USA), EDTA-retrieval, Final dilution: 1:50
***ZO-1***	fw: TCTGATCATTCCAGGCACTCGC rv: CCACATCTGGTTGCCAACTTGG 225 bp, 58°C	polyclonal rabbit anti-ZO-1 antibody No. 61–7300, (Invitrogen, Carlsbad, CA, USA, Protease retrieval, Final dilution: 1:30
***ZO-2***	fw: AGAGGACACGCCGAGCAGATTG rv: TCCCGACATCATTGCCACCAG 272 bp, 60°C	polyclonal rabbit anti-ZO-2 antibody No. 71–1400, (Invitrogen, Carlsbad, CA, USA, EDTA retrieval, Final dilution: 1:150
***β-Actin***	fw: CATGCCATCCTGCGTCTGGACC rv: ACATGGTGGTGCCGCCAGACAG 400 bp, 60°C	not performed
***Standard protocol***	95°C: 15 min; (94°C: 30s, 58°-60°C: 30s, 72°C: 30s) 40 cycles; 72°C: 5 min	

mab: monoclonal antibody, fw: forward, rv: reverse.

### Immunohistochemical analysis of tight junctional components

Immunohistochemistry was performed using the avidin-biotin complex immunostaining method and the automated immunohistochemistry slide staining system by Ventana NexES (Ventana Medical System, Strasbourg, France) as described previously [36]. Details for antigen retrieval and primary antibodies are illustrated in Table 2. Dilutions of primary antibodies were determined using appropriate positive and negative controls. For negative controls, primary antibody was replaced by irrelevant rabbit IgG that did not reveal specific signals (data not shown). Immunoreactivity was assessed in 5 representative high power fields (Zeiss Axioskop 50) of each sample by one blinded pathologist (DK). For semiquantitative assessment an adaptation of a score system originally described by Remmele et al. was applied [42]. Briefly, staining intensity ([SI], 1 = weak, 2 = moderate, 3 = strong) and the percentage of positive cells ([PPC], 1 = <10%, 2 = 10-50%, 3 = 51–80%, 4= > 80%) were scored semiquantitatively, resulting in an immunoreactive score [IRS = SI x PC] between 0 and 12. Furthermore, a score for membranous staining (0 = none, 1 = weak, 2 = moderate, 3 = strong/complete) was added resulting in a possible maximum of 15 points for each sample.

### Statistical analysis

Data are expressed as absolute number, relative proportion, median + range or mean ± standard deviation (SD) if not stated otherwise. Since the majority of data sets revealed skewed distribution, non-parametric Kruskal-Wallis test were applied for all comparisons made among the three groups (controls, NERD and ERD). If significant differences were identified (P < 0.05), post hoc analyses for pairwise comparisons between groups were performed using Mann–Whitney *U* test for gene expression analysis and immunohistochemistry. Age and histopathological parameters were analyzed by ANOVA and *T* test; frequencies by chi-square test. Non-parametric correlation analysis was performed by Spearman’s rank correlation test to investigate potential association between gene expression levels and histomorphological changes. Correlation analyses were performed in explorative manner only; adjustment for multiple comparisons was not performed. All tests were applied two-sided with a level of significance of P < 0.05.

## Results

### Patients and GERD-specific histomorphological changes

The three groups as well as the subgroups (randomly selected for immunohistochemistry) did not differ with respect to age and *H. pylori* status (Table 1). Histomorphological alterations are shown in Table 3. Activity and chronicity scores in esophageal mucosa were slightly higher in patients with NERD or ERD *vs.* controls without reaching significance. Basal cell hyperplasia, dilated intercellular spaces and elongation of papilla were significantly increased in both endoscopic entities (Table 3).

**Table 3:** Histopathological parameters

	**Controls**	** NERD**	** ERD**	**P-value One way ANOVA**
***Activity***	0 ± 0	0.23 ± 0.54	0.22 ± 0.41	n.s.
***Chronicity***	0.72 ± 0.54	0.97 ± 0.51	1.05 ± 0.67	n.s.
***Basal cell hyperplasia [BSH]***	0.52 ± 0.59	1.11 ± 0.63	1.42 ± 0.84	<0.001
***Papillary elongation [PE]***	1.32 ± 0.80	1.71 ± 0.86	2.07 ± 0.85	<0.001
***Dilated intercellular spaces [ICS]***	0.72 ± 0.68	1.49 ± 1.01	2.10 ± 0.13	<0.001

Parameters were scored semiquantitatively as described in “Patients and Methods”. Data are presented as mean ± sd.

### Upregulation of tight junction-related proteins in esophageal mucosa in context to the presence of gastroesophageal reflux disease

**Figure 1:**
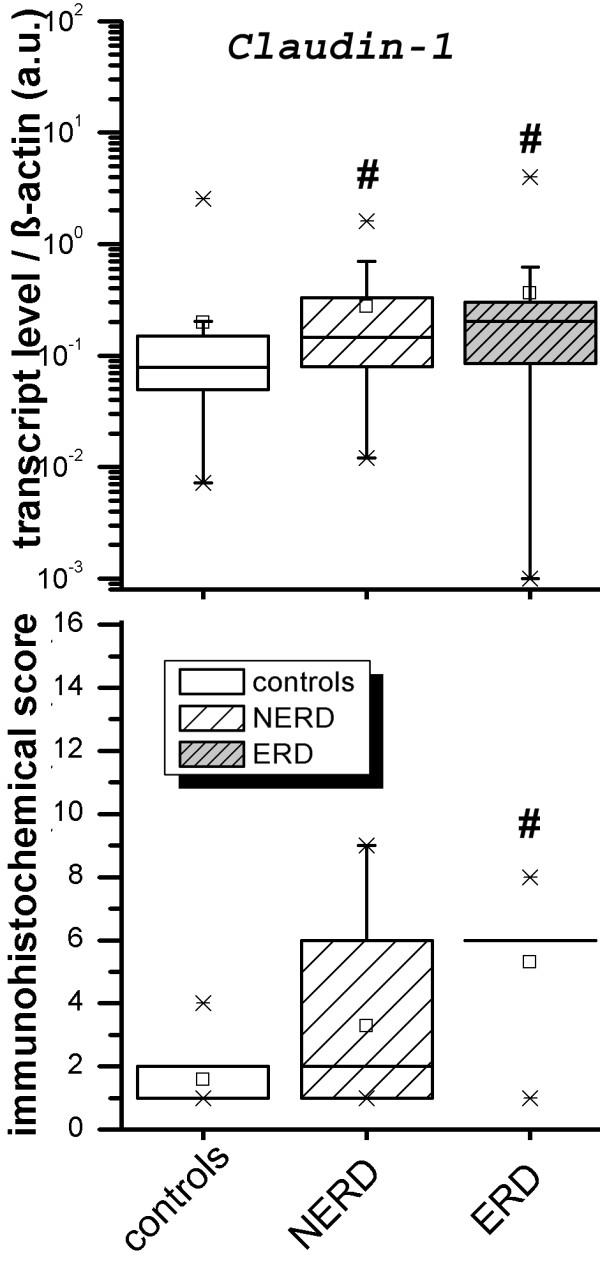
**Expression of Claudin-1 in the esophageal mucosa of patients with NERD and ERD.** The upper panel presents data of RT-PCR analysis; the lower panel illustrates immunohistochemical scores. Data are shown as boxplots illustrating 25, 75 percentile, median and 5–95 range. Note that due to skewed data distribution Claudin-1 (ERD) is presented as line only. Significant differences compared to controls are marked by a star (#); further details are presented in Tables 4A, and B.

As exemplarily demonstrated in figure 1, Claudin-1 transcript and protein levels in esophageal mucosa were significantly increased in patients with ERD, while a weaker increase was noted in NERD compared to controls. Corresponding data for the other four genes (Claudin-2, ZO-1, ZO-2; Occludin) including those of Claudin-1 are summarized in tables 4A and 4B. Claudin-2 had a similar expression pattern as Claudin-1, and both ZO-1 and ZO-2 showed a tendency to higher transcript levels in ERD and NERD (*P*-values <0.07, Table 4A). In addition to the upregulation in context to controls, both transcript levels and immunohistochemical scores of Claudin-1 were significantly higher in patients with ERD compared to those with NERD (Figure 1).

**Table 4:** Expression of tight junction-related components in esophageal mucosa in patients with GERD

**Panel A** **Transcript level**	**Gene expression/ß-actin (a.u.) median (range)**	**Change*vs.*** **controls (x-fold)**	*P*-values (* Kruskal-Wallis; posthoc: Man Whitney*U*test)
**Occludin**			0.098*
*controls*	0.041 (0.0086 - 0.38)		
*NERD*	0.060 (0.0052 - 0.57)	1.46	n.a.
*ERD*	0.035 (0.0026 - 1.09)	0.85	n.a.
**Claudin-1**			0.0097*
*controls*	0.078 (0.0072 - 2.54)		
*NERD*	0.15 (0.012 - 1.6)	1.92	**0.016**
*ERD*	0.20 (0–4.1)	2.56	**0.0032**
**Claudin-2**			0.0027*
*controls*	0.000038 (0–0.003)		
*NERD*	0.0002 (0–0.019)	5.26	**0.0041**
*ERD*	0.000083 (0–0.021)	2.18	0.11
**ZO-1**			0.069*
*controls*	0.0060 (0.0012 - 0.073)		
*NERD*	0.0081 (0–0.067)	1.35	n.a.
*ERD*	0.0077 (0.0015 - 0.21)	1.28	n.a.
**ZO-2**			0.061*
*controls*	0.011 (0.0022 - 0.038)		
*NERD*	0.019 (0.002 - 0.27)	1.72	n.a.
*ERD*	0.021 (0.0019 - 0.59)	1.9	n.a.
**Panel B** **Protein level**	**IHC score** **median (range)**	**Change (x-fold)** * vs.* controls	*P*-values (* Kruskal-Wallis; **posthoc: Man Whitney*U*test)**
**Occludin**			0.02*
*controls*	3 (1 – 9)		-
*NERD*	6 (1 – 15)	2.0	***0.026***
*ERD*	8 (1 – 12)	2.7	***0.012***
**Claudin-1**			0.014*
*controls*	1 (1 – 4)		-
*NERD*	2 (1 – 9)	2.0	***0.14***
*ERD*	6 (1 – 8)	6.0	***0.0004***
**Claudin-2**			0.0057*
*controls*	4 (1 – 9)		-
*NERD*	6 (1 – 12)	1.5	0.28
*ERD*	9 (4 –15)	2.25	***0.0025***
**ZO-1**			0.62*
*controls*	2 (0 – 6)		-
*NERD*	4 (1 –12)	2.0	n.a.
*ERD*	3 (0 – 3)	1.5	n.a.
**ZO-2**			0.58*
*controls*	4 (0 – 10)		-
*NERD*	4 (0 – 8)	1.0	n.a.
*ERD*	1 (0 – 8)	0.25	n.a.

Transcript levels are shown in relation to controls (Panel A). Immunohistochemical scores are illustrated similarly (Panel B). Statistical analyses for both datasets were done first by non-parametric Kruskal-Wallis (*P-value italic style*); if significant post-hoc analysis was done by Mann Whitney *U* test. Significant changes are demonstrated by bold letters. n.a.: not applicable.

**Figure 2:**
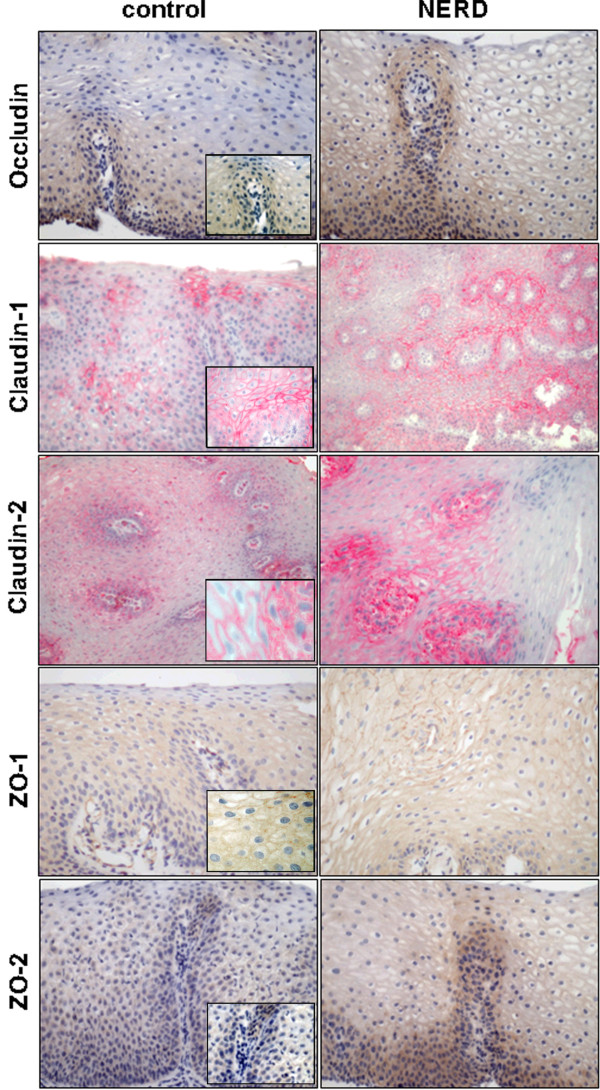
**Immunohistochemical stainings of tight junction-related proteins in esophageal mucosa.** Occludin, Claudin-1, -2 and ZO-1,-2 are displayed by brown or red staining, respectively. Panels illustrate representative staining for controls and samples obtained from patients with NERD. Immunohistochemical staining was observed in the esophageal squamous epithelium mainly at the basal and suprabasal zone. Claudin-1/2 and ZO-1 showed partly a membranous staining. (Zeiss Axioskop 50; camera: Nikon coolpix 990).

In general, higher transcript levels were accompanied by higher immunohistochemical scores for most proteins. In addition to these quantitative changes in gene expression, different patterns of protein distribution within the cell compartment and within different mucosal layers were noted (Figure 2). In controls, the expression of tight junction-related proteins was mainly observed in the basal epithelial layers and in a cytoplasmatic pattern. In GERD, expansion of protein expression to the suprabasal und spinous epithelial layers was observed. Furthermore, expression of Claudin-1, Claudin-2 and ZO-1 was partly membrane-associated with a stronger intensity in GERD compared to controls.

### Increased gene expression of tight junction-related molecules (transcript level) does not correlate with histomorphological changes in esophageal mucosa

**Figure 3:**
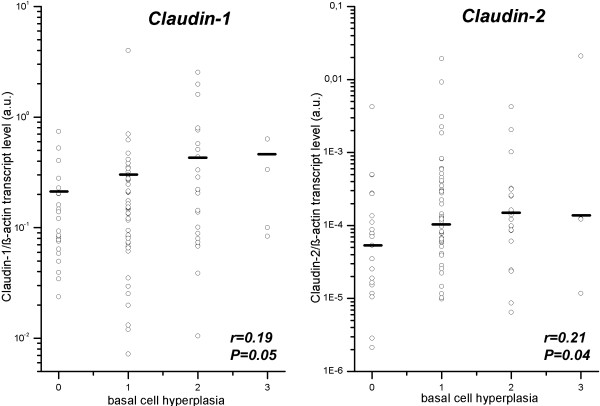
**Correlation of Claudin-1 and Claudin-2 with histomorphological changes in esophageal mucosa.** Panels illustrate correlations between transcript levels and histomorphological alterations as indicated. Data are shown as open dot plots; medians are presented filled dot. Non-parametric correlation analysis was performed by Spearman’s rank correlation test; *P values* are presented in figure. Detailed data of other correlations are presented in Tables 5A, and 5B.

In order to study potential correlations between gene expression levels (transcript level) and the degree of histopathological alterations, all three groups were analyzed together in the first step. As exemplarily illustrated in figure 3, gene expression levels of Claudin-1 and Claudin-2 marginally correlated with the degree of basal cell hyperplasia, but not with dilated intercellular spaces and length of papilla (data not shown). Since most analyses were negative, these data are summarized in Table 5A for all five genes. Since basal cell hyperplasia revealed some even weak correlations in the complete study cohort, these correlation analyses were performed again for all three groups individually and for patients with GERD (NERD + ERD) combined. Only 3 out of 20 subanalyses revealed marginally significant correlations (without adjustment for multiple comparisons), two of those were identified in controls (data summarized in Table 5B).

**Table 5:** Correlation between GERD-specific histopathological alterations and gene expression level of tight junction-related genes (transcript level)

**Panel A: All samples**	***Occludin***	***Claudin-1***	***Claudin-2***	***ZO-1***	***ZO-2***
*Activity*	n.s.	r = 0.23	n.s.	n.s.	n.s.
		*P* = 0.07			
*Chronicity*	n.s.	n.s.	n.s.	n.s.	n.s.
*Basal cell hyperplasia*	n.s.	r = 0.19	r = 0.21	r = 0.22	n.s.
		*P* = 0.05	***P*** **= 0.04**	***P*** **= 0.03**	
*Elongation of papilla*	n.s.	n.s.	n.s.	n.s.	n.s.
*Dilated intercellular space*	n.s.	n.s.	n.s.	n.s.	n.s.
**Panel B: Basal cell hyperplasia**	***Occludin***	***Claudin-1***	***Claudin-2***	***ZO-1***	***ZO-2***
*Controls*	r = 0.47	n.s.	n.s.	r = 0.36	r = 0.42
	***P*** **= 0.02**			*P* = 0.08	***P*** **= 0.04**
*NERD*	r = 0.36	n.s.	n.s.	n.s.	n.s.
	***P*** **= 0.03**				
*ERD*	n.s.	n.s.	n.s.	n.s.	n.s.
*GERD (ERD + NERD)*	n.s.	n.s.	n.s.	n.s.	n.s.

Panel A: The histopathological scores (Table 3) were correlated with gene expression levels (transcript levels, Table 4) for each gene individually in the combined study cohort (n = 110). Data (r-, and P-values) represent potential correlations between these parameters (n.s. = not significant). Panel B: Since basal cell hyperplasia demonstrated significant correlations in global analysis (Panel A), this parameter was further analyzed by correlating expression values for all five genes with basal cell hyperplasia within each group and for patients with GERD individually as identified in table.

In addition to the correlation based on transcript levels (Figure 3, Table 5), correlation analysis between protein expression levels (immunohistochemical scores, Table 4B) and histopathological alterations (Table 3) was performed. Here, only one significant correlation (between Claudin-1 and activity of inflammation, r = 0.51, P < 0.01) was identified (data not shown).

## Discussion

In this study, we demonstrated (I) distinct expression patterns of five genes encoding for proteins involved in the formation of tight junctions in esophageal mucosa. In particular Claudin-1 in ERD and to lesser extent Claudin-2 was expressed at higher levels in patients with GERD. In contrast, ZO-1, ZO-2, and Occludin were not affected by the presence of GERD. (II) In general, altered gene expression of Claudin-1/-2 did not correlate with the degree of histomorphological changes in the esophageal mucosa of patients with GERD.

Tight junctions are composed of transmembrane proteins such as Occludin, 24 Claudins, several junctional adhesion molecules (JAMs) with different isoforms, E-Cadherin as well as cytosolic binding partners [43,44]. The selection of the five genes studied was based on functional aspects. Occludin is critical for the formation of tight junctions in most tissues [45]. Claudin-1 is one of the numerous Claudins that seals intercellular space leading to higher barrier function [46], while Claudin-2 is the only pore-forming member of this family resulting in increased permeability [47]. Zonula occludens (ZO)-1 and-2 are cytosolic partners of tight junctions in most epithelial surfaces [48,49]. The selected genes present important components of the tight junctional complex, and were considered to allow assessment about alterations of tight junctions in relation to GERD. A comprehensive analysis concerning the general expression pattern of other junctional proteins was not performed.

Recently, several studies demonstrated characteristic histopathological alterations in esophageal mucosa of patients with GERD and a proinflammatory response including the activation of related pathways such as NFκB, PAR-2, ROS and iNOS [50,51]. Several *in vitro* and animal studies have provided evidence that incubation of esophageal mucosa or squamous cell lines either with acidified media with/without bile acids or proinflammatory cytokines can provoke changes in transepithelial electric resistance and increased transepithelial permeability [52-55]. Notably, several studies demonstrated a cytokine-mediated change of tight junction-related molecules in various cell models. For instance, IL-6 markedly induces Claudin-2 expression *via* MEK and PI3K signaling leading to increased tight junction permeability [56]. In a rabbit model of GERD, elevated IL-6 expression correlated with induction of several tight junction-related proteins (Claudin-1, Occludin, JAM-1, ZO-1) [57] and altered the motogenic activity of smooth muscle cells [58].

All together, there is sufficient data showing that the exposure of mixed gastric or gastroduodenal refluxate causes altered esophageal epithelial barrier function, inflammation and cellular damage, although the timely order of these processes is a matter of debate [20]. As today, it is well accepted that impaired epithelial barrier function of the distal esophagus presents a major pathophysiological process in GERD.

This study shows an upregulation of tight junction-related proteins in relation to ERD and NERD in mucosal samples. In particular, Claudin-1 and Claudin-2, though mediating opposite functionally effects, were induced, while cytoplasmic adapters and Occludin were rather unchanged in relation to controls. The higher expression of Claudin-1 (both on transcript and protein level) was the only significant difference identified between patients with ERD and NERD. The fact that all other identified changes were similar between NERD and ERD supports the concept of similar pathophysiological mechanisms between both diseases. Unexpectedly, these changes did not correlate with histomorphological alterations, in particular with dilated ICS in esophageal mucosa. This finding is in contrast to the recently identified correlation between histopathological alterations, in particular basal cell hyperplasia, and elevated gene expression of desmosomal proteins [39]. In this study, few borderline correlations were found for basal cell hyperplasia and some genes only, but notably these findings were mostly restricted to reflux-negative controls, whereas patients with GERD did not reveal significant correlations between histopathological alterations and transcript levels of the five genes. Since correlation analyses were performed in an explorative way (without adjustment for multiple comparison), the few significant correlations (with borderline significance) do not support a general role of these findings for the pathophysiology of GERD. Taken into consideration this limitation and the fact that that the overall majority of our comparisons (17 out of 20) revealed no correlations, we conclude that our data do not give evidence for an association between the gene expression of the five genes studied and the histopathological changes in our study groups. It is well known that extent of basal cell hyperplasia reflects proliferative status of esophageal mucosa [9]. Since the identified correlations between gene expression levels and basal cell hyperplasia were mostly restricted to controls, it is unlikely that elevated Claudin-1 levels in ERD reflect tissue repair in context to mucosal damage caused by refluxate in these patients. Since we and others demonstrated more severe histomorphological alterations in ERD than NERD, the overall consistent changes of the 5 genes and their corresponding proteins in both diseases seem to be of limited relevance to the mucosal integrity and function. Furthermore, it is notable that some of the stainings revealed not the typical membrane-restricted expression pattern as demonstrated for these tight junction-related molecules im most gastrointestinal tissues [59,60]. However, cytoplasmic or diffuse membranous expression patterns have been identified for Claudin-2 [60] and ZO-1 [61] in human gastrointestinal tissue and for Claudin-1 in esophageal mucosa of rat [62]. Occludin staining pattern or expression in esophageal mucosa differs frequently also from those identified in gastric or intestinal mucosa [60,63]. Overall, the subcellular distribution of the 5 tight junction-related proteins seems to differ partially from those identified in columnar-lined epithelium. However, the study was not aimed to analyze the subcellular distribution pattern of the molecules in esophageal mucosa on the subcellular level. The presence of appropriate negative and positive control stainings in other tissues, and the good concordance between expression data on transcript and protein level in general provide further indirect evidence for the specificity of immunohistochemical stainings.

Based on the descriptive study design, it remains open whether the altered gene expression levels of Claudin-1 and −2 contribute to GERD pathophysiology or merely are markers for the existing disease. Furthermore it is notable that the majority of patients received GERD medications (PPI, H2RA) in the past before entering study. Even a stop of at least 2 weeks was mandatory to enter the study, we can not exclude that the effects of long-term therapy in the past or the changes induced by the 2-week stop of medication (e.g. acid rebound) [64] could have affect the expression of the five genes studied. Another limitation is the assessment of protein expression by an immunohistochemical score that can be done semiquantitatively at best. Besides this methodological aspect, posttranscriptional regulatory mechanisms can lead to different findings between gene expression analysis performed on transcript and protein levels. But as mentioned above, overall we observed a good concordance between both levels even not all significant findings were confirmed by both methodologies. Since we studied five selected components of tight junction complexes in GERD only, general conclusions can not be made. Assessment of other tight junction related molecules (e.g. Claudins, JAMs, Tricellulin) [44,46,65] in regard to GERD needs to be performed.

## Conclusions

In summary, this study demonstrates a partial upregulation of tight junction-related components, in particular Claudin-1, in relation to GERD. Since identified molecular changes do not correlate with histomorphological alterations in general, a major role of Claudin-1 as of the other four tight junction-related proteins in the pathogenesis of GERD can not be concluded from our study.

## Abbreviations

BE: Barrett’s esophagus; ERD: Erosive reflux disease; NERD: Nonerosive reflux disease; GEJ: Gastroesophageal junction; GERD: Gastroesophageal reflux disease; H2RA: Histamine-receptor antagonist; H. pylori: Helicobacter pylori; ICS: Intercellular spaces; INR: International normalized ratio; iNOS: Inducible nitro oxygen synthetase; JAM: Junctional adhesion molecule; NFκB: Nuclear factor kappa B; PAR-2: Protease activated receptor-2; PPI: Proton pump inhibitor; RDQ: Reflux disease questionnaire; ROS: Reactive oxygen species; RT-PCR: Reverse transcription polymerase chain reaction; ZO: Zona occludens.

## Competing interests

The authors declare that they have no competing interest concerning the content of this article.

## Authors’ contributions

KM, PM and TW designed the study. KM, LCF enrolled the majority of patients. AK provided clinical data. TW coordinated and performed laboratory work. DK and AR provided histopathological and immunohistochemical data. TW and SK performed statistical analysis. The manuscript was drafted by TW, AK, DK, and reviewed for important intellectual content by KM and PM. All authors read and approved the final manuscript.

## Pre-publication history

The pre-publication history for this paper can be accessed here:

http://www.biomedcentral.com/1471-230X/12/128/prepub
